# Evolution of Microstructure and Performance in Polyacrylonitrile Precursor Fibers: A Comparison of Spinning Processes

**DOI:** 10.3390/polym17182504

**Published:** 2025-09-17

**Authors:** Liang Cao, Lili Zhang, Zhenbo Zhao, Shaowei Wang, Zhaowei Li, Deqi Jing, Shouchun Zhang

**Affiliations:** 1 Research Center of Advanced Thermoplastic Composites Engineering, Institute of Coal Chemistry, Chinese Academy of Sciences, Taiyuan 030001, China; 2 Center of Materials Science and Optoelectronics Engineering, University of Chinese Academy of Sciences, Beijing 100049, China; 3 Shanxi Key Laboratory of Carbon Materials, Institute of Coal Chemistry, Chinese Academy of Sciences, Taiyuan 030001, China

**Keywords:** polyacrylonitrile fiber, wet spinning, dry-jet wet spinning, structural evolution

## Abstract

The microstructure of polyacrylonitrile (PAN) precursor fibers has a profound influence on the performance of carbon fibers and depends on the spinning processes and processing conditions. This study compared the evolution of the microstructures and performance of PAN fibers between the wet-spinning and dry-jet wet-spinning processes, utilizing scanning electron microscopy, small/wide-angle X-ray scattering, dynamic mechanical analysis, and single-fiber tensile testing. Both spinning processes promoted the oriented alignment of microfibrils and fibrils, improved the crystal arrangement and molecular regularity, and facilitated the transition from a two-phase (crystalline/amorphous) structure to a single-phase structure, thereby gradually improving the fibers’ elastic character and mechanical properties. However, wet-spun fibers exhibited inherent defects (skin-core structure and large voids), which caused surface grooves, radial mechanical heterogeneity, and low breaking elongation during post-spinning. In contrast, dry-jet wet-spun fibers initially had a smooth surface and a homogeneous radial structure, which evolved into well-oriented, radially homogeneous structures during post-spinning. Furthermore, the dry-jet wet-spinning process produced greater increases in crystallinity (46%), crystal size (258%), and orientation index (146%) than the wet-spinning process did. The dry-jet wet-spinning process’s superiority in forming and optimizing the fiber microstructure gives it greater potential for producing high-quality PAN precursor fibers.

## 1. Introduction

Polyacrylonitrile (PAN)-based carbon fibers are excellent reinforcement materials for composites, which have a wide range of applications in aircraft [1], automobile [2,3], wind turbine blades [4,5,6], high-pressure hydrogen storage vessels [7], and other fields. The preparation of PAN-based carbon fibers involves a series of complex stages, including precursor fiber spinning, pre-oxidation treatment, and subsequent carbonization [8,9,10,11]. The mechanical properties (such as strength and modulus) of carbon fibers mainly depend on their crystalline structure and the defects [12,13]. Wu et al. [13] found that nitrogen atoms with different forms can increase defects and form imperfect graphite microcrystals by distorting the graphite sheets, ultimately leading to a decrease in tensile strength. Kunzmann et al. [14] further pointed out that the tensile strength of carbon fibers decreased as the effective pore area and pore aspect ratio on the fiber surface increased. Meanwhile, they also revealed a genetic correlation between the nanopore structure of carbon fibers and their corresponding PAN precursor fibers. Furthermore, the research conducted by Gao and Wang [15] demonstrated that precursor fibers possessing an ordered, and closely arranged, fibrillar structure facilitate the preparation of high-performance carbon fibers characterized by a highly ordered graphite arrangement. These studies indicate that the microstructure of precursor fibers can affect the crystal structure and defects of the carbonization products through hereditary effects, which play a crucial role in determining the performance of carbon fibers.

The microstructure of PAN precursor fibers is formed and developed during the spinning process [16]. Currently, commercial carbon fibers are primarily produced using wet-spinning or dry-jet wet-spinning techniques [11,12]. The main difference between these methods lies in the mechanism by which nascent fibers are formed in coagulation baths. In wet spinning, the polymer solution is directly extruded through a spinneret and immersed in the coagulation bath. In contrast, dry-jet wet spinning involves the fluid filament passing through an air gap before entering the coagulation bath [11,12]. This process can reduce the surface defects and obtain smoother fibers [12,17]. However, regardless of the method used, the nascent fibers lack the necessary qualities to serve as precursors for high-performance carbon fibers as they contain numerous void defects and residual solvents [18,19]. Therefore, the microstructure of fibers must be optimized through post-spinning processes, which include washing, hot-water stretching, oiling, drying densification, and steam stretching [11,20,21]. In addition, process parameters are crucial to the microstructural evolution of the fibers [12,21,22]. Gao et al. [23] found that increasing the drawing ratio during the coagulation bath stage of dry-jet wet spinning improves the regularity of the microfibril alignment and the radial homogeneity of the fibers, thereby enhancing the mechanical properties of the PAN precursor fibers. The evolution of the microfibril network during the spinning process undergoes three stages: plastic deformation, fragmentation of lamellae, and regular arrangement [22]. Moskowitz et al. [24] noted that steam stretching significantly promoted the increase in crystal size. Under a specific set of process parameters (the lowest jet stretch and the highest steam stretching ratio), the PAN precursor fibers exhibited the strongest mechanical properties, the largest crystal size and orientation, and the highest thermostability [24]. In the preparation of PAN precursor fibers, simulations have become an important tool for studying the relationship between process parameters and fiber properties. Oh et al. [25] combined numerical simulations with experimental data to investigate the diffusion coefficients of solvent and non-solvent during the coagulation process in wet spinning. Wang et al. [26] analyzed the spinnability of PAN solution in dry-jet wet spinning through spinning dynamics simulation, and their results indicated that, under the premise of ensuring smooth extrusion, appropriately lowering the spinning temperature or increasing the mass flow rate to raise the pressure drop can help avoid poor spinnability issues such as filament adhesion.

At present, our understanding of the microstructural characteristics of commercial PAN precursor fibers produced by dry-jet wet-spinning and wet-spinning techniques remains insufficient. Typically, the surfaces of wet-spun PAN precursor fibers present grooves, while the surfaces of dry-jet wet-spun fibers are relatively smooth. However, the other microstructural differences (e.g., radial structure homogeneity, void structure, and crystalline structure) and their evolution mechanisms between the two types of PAN fibers have not been systematically compared in previous studies. These microstructural features may significantly influence the structure and properties of the final carbon fibers. Therefore, conducting comprehensive research on the microstructural characteristics of PAN precursor fibers and their evolution during the spinning process and clarifying the similarities and differences between the two spinning processes will provide valuable guidance to produce high-performance carbon fibers.

In this study, we conducted a comparative investigation of the microstructural evolution and mechanical property variations in PAN fibers between the wet-spinning and dry-jet wet-spinning processes. To track structural evolution, the PAN fibers were collected from each key stage of pilot-scale production lines representing both spinning methods. The morphological structures, voids, crystal structure, and phase transitions of the fibers were analyzed. In addition, the mechanical properties of the fibers were assessed through single-fiber tensile testing. These results help researchers and manufacturers understand the microstructural and mechanical properties evolution of the two spinning processes, thus providing valuable guidance on selecting and optimizing the spinning process.

## 2. Materials and Methods

### 2.1. Preparation of Fiber Samples

**Figure 1:**
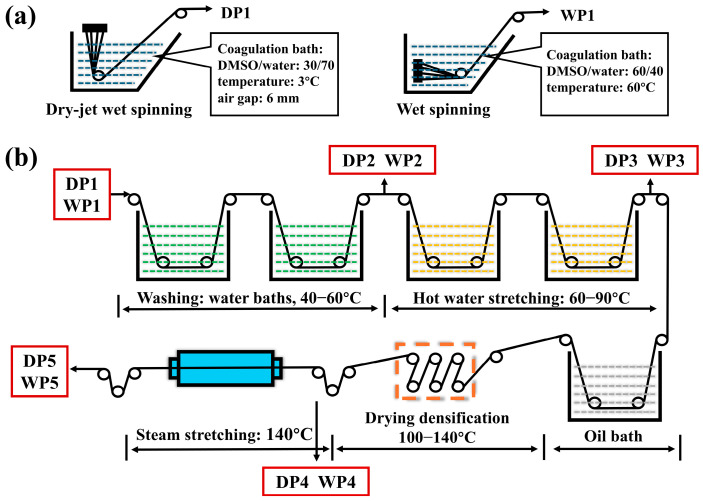
Sketch of spinning process of two PAN fiber: (**a**) the coagulation bath of dry-jet wet-spinning and wet-spinning method and (**b**) post-spinning process of the two PAN nascent fibers.

The PAN copolymer was synthesized via free radical solution polymerization in dimethyl sulfoxide (DMSO) using acrylonitrile and itaconic acid (2 wt%) as comonomers and 2,2′-azobisisobutyronitrile (AIBN) as the initiator, and the spinning solution (20% polymer content) is obtained through degassing and precise filtration. Two types of PAN fibers were then produced by wet-spinning and dry-jet wet-spinning processes on a pilot production line and each process utilized respective optimized and matched processing conditions, as illustrated in Figure 1. Initially, the spinning solution at 60 °C was fed into the spinneret assembly by a gear pump. The nascent fibers collected from the coagulation baths of the dry-jet wet-spinning process and wet-spinning process were designated as DP1 and WP1, respectively (Figure 1a). In the coagulation bath, the drawing ratio for dry-jet wet spinning is 2, while that for wet spinning is 0.7. Subsequently, the fibers underwent a post-spinning process and were collected after washing, hot-water stretching, drying densification, and steam stretching, which were assigned as P2, P3, P4, and P5 in sequence (Figure 1b). Notably, samples from the dry-jet wet-spinning series were prefixed with “D”, while samples from the wet-spinning series were prefixed with “W”. The applied total drawing ratio was 20 for the dry-jet wet-spinning process and 9.8 for the wet-spinning process, finally yielding precursor fibers (DP5 and WP5) with similar diameters.

### 2.2. Characterization

The scanning electron microscopy (SEM) (JSM-7900F, JEOL Ltd., Tokyo, Japan) was employed to observe morphological structures of the PAN fibers, including both the surface and cross-section, after a thin layer of platinum was applied through sputter-coating. The wide-angle X-ray scattering (WAXS) analysis was conducted using a 2D X-ray scatterer (Nano-inXider, Xenocs, Grenoble, France) with Cu Kα radiation (wavelength *λ* = 0.154 nm), and the sample-to-detector distance for WAXS was 79 mm. To analyze the crystal size and crystallinity of PAN fibers, the 1D scattering curves were extracted from the 2D WAXS pattern and fitted using the Pearson VII function. The crystal plane spacing (*d_hkl_*), crystal size (*L_hkl_*), and crystallinity (*X_C_*) were calculated using Equations (1)–(3) [24]:

(1) $$\begin{array}{c} d_{\mathrm{hkl}}=\frac{\lambda }{2\sin\theta_{\mathrm{hkl}}} \end{array}$$

(2) $$\begin{array}{c} L_{\mathrm{hkl}}=\frac{0.89\lambda }{\beta_{\mathrm{hkl}}\cos\theta_{\mathrm{hkl}}} \end{array}$$

(3) $$\begin{array}{c} X_{C}=\frac{A_{c}}{A_{t}}\;\times \;100\% \end{array}$$

Here, *λ* is the wavelength of the X-ray, which is 1.54 Å. The term *β_hkl_* denotes the full width at half maximum (FWHM) of the diffraction peak, while *θ_hkl_* refers to the Bragg angle associated with that peak. *A_c_* represents the area of the crystallization peak, and *A_t_* signifies the area encompassing all peaks. The orientation of the crystallites within the fiber was measured using an X-ray diffractometer (D8-Advance, Bruker, Karlsruhe, Germany) operating at 40 kV and 50 mA, utilizing Cu Kα radiation (wavelength *λ* = 0.154 nm). The azimuthal intensity data were extracted using the azimuthal intensity integral along the circumference of the diffraction ring (as shown in Figure S11). The crystal orientation index (*OI*) of the fibers was evaluated according to the azimuthal distribution width (H) of the diffraction ring, as described in Equation (4) [27]:

(4) $$\begin{array}{c} OI=\frac{180-H}{180}\times 100\% \end{array}$$

Here, the *H* is the FWHM of the azimuthal scan, which is determined through fitting with a Pearson VII function. The void structure of the fiber was estimated by small-angle X-ray scattering (SAXS), conducted at the 1W2A beamline of the Beijing Synchrotron Radiation Facility (BSRF). The incident X-ray wavelength was 1.54 Å, and the sample-to-detector distance was fixed at 1650 mm. The SAXS detector utilized was a Mar165 CCD. The width of voids (*L_3_*) was determined using Equation (5) proposed by Shioya and Takaku [28].

(5) $$\begin{array}{c} L_{3}=2\frac{\int_{0}^{\infty }I\left(q\right)dq}{\int_{0}^{\infty }I\left(q\right)qdq} \end{array}$$

The length (*L*) and orientation angle (*B_eq_*) of voids along the fiber axis can be obtained through the Ruland streak method [29,30], as follows:

(6) $$\begin{array}{c} B_{\pi /2}^{2}s^{2}=B_{eq}^{2}s^{2}+\frac{1}{L^{2}} \end{array}$$

where *s* = *q*/2*π*, and the *L* and *B_eq_* can be obtained by linearly fitting the plot of *s*^2^*B_π/2_*^2^ versus *s*^2^. The dynamic mechanical analysis (DMA) was conducted using the NETZSCH DMA 242. The fiber tows were tested in tension mode at a frequency of 1 Hz in a nitrogen atmosphere. The testing temperature range was from 25 °C to 200 °C, with a heating rate of 3 °C/min. Mechanical properties were assessed by single-fiber tensile testing using an electronic single-fiber strength machine (GY001E, Fang Yuan Instrument, Wenzhou, China) with an initial length of 20 mm and a tensile rate of 10 mm/min.

## 3. Results and Discussion

### 3.1. Morphology

**Figure 2:**
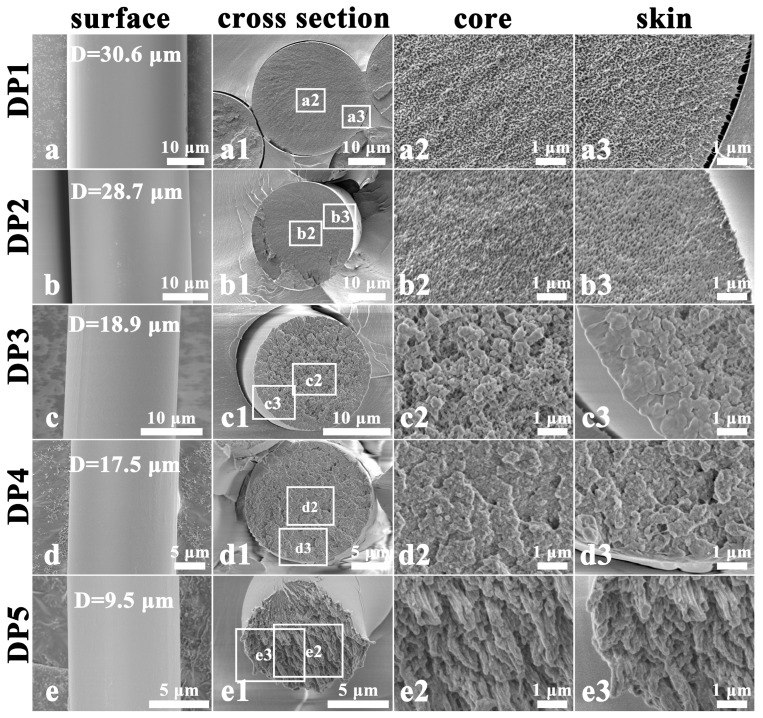
SEM images of the surface and cross-section of PAN fibers at different stages of the dry-jet wet-spinning process: surface (**a**–**e**), cross-section (**a1**–**e1**), core region at 20,000× magnification (**a2**–**e2**), and skin region at 20,000× magnification (**a3**–**e3**). “D” represents the average diameter of the fibers.

Figure 2 illustrates the surface and cross-section morphology of PAN fibers at different stages of the dry-jet wet-spinning process. After the coagulation bath stage, the PAN fiber (DP1) displays a smooth surface, a relatively homogeneous microfibril network, and a porous structure (Figure 2(a1–a3)). As the PAN fluid passes through an air gap before entering the coagulation bath, a smooth gelatinized thin layer is formed on the fiber surface due to small amount of solvent (DMSO) evaporation and a drop in temperature [31]. This layer reduces the diffusion rate of water (H_2_O) within the fiber due to its hydrophobic property, thereby delaying coagulation and promoting the formation of a homogeneous microfibril network [31]. It is worth noting that the smooth surface characteristic is continuously retained in the subsequent processes (Figure 2a–e). After the washing stage, the microfibril network (DP2) slightly shrinks, and the size of the voids decreases (Figure 2(b1–b3)). Following the hot-water stretching stage, the microfibril network (DP3) undergoes significant aggregation, simultaneously forming a dense skin layer (Figure 2(c1–c3)). This phenomenon suggests that the radial shrinkage originates from the skin layer, which is caused by the uneven radial distribution of stretching stress within the fibers [20]. After the drying densification stage, aggregated microfibrils (DP4) further merge, and the number of voids reduced significantly (Figure 2(d1–d3)) due to water removal. In addition, the cross-section of DP4 exhibits a relatively homogeneous morphological structure, indicating that the drying densification process can minimize the differences between the skin and core regions caused by the void structure. After steam stretching, the cross-section of DP5 displays highly oriented and uniformly distributed fibrils with diameters of approximately 100–500 nm (Figure 2(e1–e3)). In this process, the synergistic action of the tensile field and the plasticizing effect of water molecules enables the macromolecular chains to slide and align along the fiber axis, thereby promoting the orientation of the fibrils. Furthermore, the post-spinning process facilitates the fiber diameter (D) to reduce from 30.6 μm to 9.5 μm. This reduction in diameter typically not only decreases the density of structural defects but also effectively inhibits the propagation of microcracks through the closely arranged and highly oriented fibrillar structure, thereby enhancing the mechanical properties of the fibers [21].

**Figure 3:**
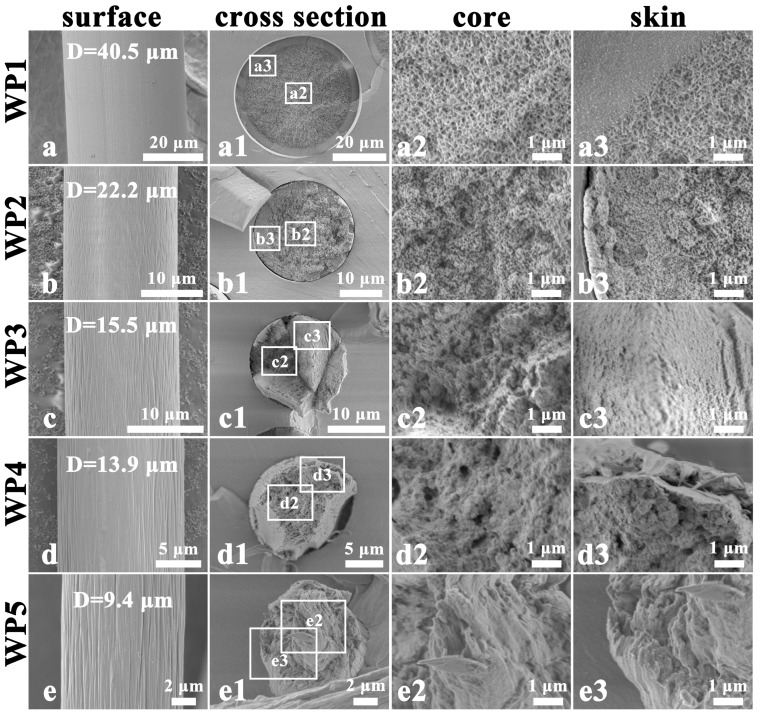
SEM images of the surface and cross-section of PAN fibers at different stages of the wet-spinning process: surface (**a**–**e**), cross-section (**a1**–**e1**), core region at 20,000× magnification (**a2**–**e2**), and skin region at 20,000× magnification (**a3**–**e3**). “D” represents the average diameter of the fibers.

The surface and cross-sectional morphological evolution of wet-spun PAN fiber is shown in Figure 3. At the coagulation stage of wet spinning, WP1 exhibits a relatively smooth surface, while its cross-section shows a distinct skin-core structure that is significantly different from that of DP1 (Figure 3(a1–a3)). This radially heterogeneous structure originates from the rapid coagulation of the PAN fluid during the wet-spinning process [31,32,33]. When the spinning dope enters the coagulation bath, the outer layer of the dope initially experiences rapid phase separation due to the double diffusion of the solvent and the non-solvent. This process triggers the swift accumulation and precipitation of molecular chains in the outer region, eventually leading to the formation of a dense sheath structure. As the washing progresses, grooves begin to appear on the surface of the fiber, and the fiber diameter significantly decreases from 40.5 μm to 22.2 μm (WP2; Figure 3b). Simultaneously, the microfibril network within the fiber markedly contracts (Figure 3(b1–b3)). These phenomena are mainly attributed to the compression of large voids inside the nascent fibers under tension, which induces radial collapse of the skin layer and a significant decrease in fiber diameter. After hot-water stretching, the microfibril network (WP3) exhibits further shrinkage, with the skin layer showing a significantly denser structure relative to the core layer (Figure 3(c1–c3)). The drying densification process further integrates the microfibril network (WP4), particularly in the core region (Figure 3(d1–d3)). After steam stretching, the microfibril network evolves into a well-oriented fibril structure (WP5) due to the stress-induced rearrangement and orientation of the molecular chain. However, the fracture morphologies of the fibrils differ between the skin and core regions. Specifically, fibrils in the core exhibit pull-out behavior due to the different mechanical properties between the skin and core regions (Figure 3(e1–e3)). This observed result suggests that the skin-core structure formed in nascent fibers persists throughout the post-spinning process. The consequent stress variation between the skin and core regions causes significant radial gradient differences in the microstructure and mechanical properties of PAN precursor fibers. Additionally, both the depth and length of the surface grooves increase during the post-spinning process (Figure 3c–e). This phenomenon originates from the further radial collapse of the thick skin layer, accompanied by the shrinkage of the microfibril network under tension. Ultimately, the post-spinning process reduces the fiber diameters from 40.5 μm to 9.4 μm.

### 3.2. Void Structure

**Figure 4:**
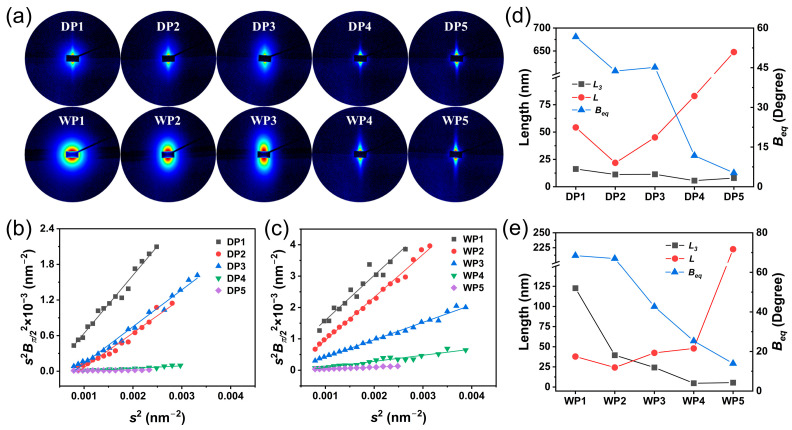
SAXS patterns of PAN fibers (**a**), the Ruland streak method fitting (**b**,**c**), the length (*L*), the width (*L_3_*), and the orientation angle (*B_eq_*) of the voids within PAN fiber at key stages of the dry-jet wet-spinning (**d**) and wet-spinning processes (**e**).

Figure 4 illustrates the evolution of the void structure at key stages in both spinning processes. At the coagulation bath stage of the spinning processes, DP1 exhibits fusiform scattering patterns, contrasting sharply with the nearly circular patterns observed in WP1 (Figure 4a). This difference reflects their distinct void structures: DP1 contains relatively narrow and elongated voids, while WP1 features wide and circular cavities. As the post-spinning process progressed, the scattering patterns gradually evolve into a needle-like shape, particularly after the drying densification stage. This transformation results from the orientation of the void structure within the fiber along the stretching direction. The void parameters of the fibers can be calculated using the Shioya-Takaku method (Equation (5)) and the Ruland streak method (Equation (6)). Figure 4b,c show the fitting for the Ruland streak method. The resulting void parameters and their variation trends during the two spinning processes are presented in Figure 4d,e. As for the two spinning processes, the orientation angle (*B_eq_*) of the voids exhibits an overall decreasing trend, indicating a continuous improvement in the alignment of the voids throughout the spinning processes.

However, the void size exhibits different evolution paths. The primary change in the void during the dry-jet wet-spinning process is axial stretching (Figure 4d). At the coagulation bath stage (DP1), the width and length of the void are 16.21 nm and 54.23 nm, respectively. After the washing stage (DP2), these values decrease to 11.23 nm and 21.87 nm, respectively, due to microfibril shrinkage induced by axial tension. As the fiber undergoes hot-water stretching (DP3), the length of the void increases significantly to 45.1 nm, while its width remains relatively unchanged. This structural transformation is primarily attributed to the rearrangement of the microfibril network under tension, which drives the oriented elongation of the void structure. Subsequent drying densification (DP4) further reduces the void width to 5.58 nm while extending the length to 82.93 nm due to the merging of microfibrils induced by water evaporation. Finally, steam stretching (DP5) results in a significant elongation of the void length to 647.58 nm through the plasticization effect of steam, indicating a highly oriented parallel structure of voids/fibrils within the PAN precursor fibers.

In contrast, the void evolution during wet-spinning process is characterized by radial shrinkage (Figure 4e). At the coagulation bath stage, the void in WP1 exhibits a width of 122.69 nm, significantly larger than its length of 37.84 nm. This discrepancy originates from the loose microfibril network and interconnected large voids in the core region (Figure 3(a2)). As the fibers are washed (WP2), the void width and length decrease to 39.46 nm and 24.25 nm, respectively, due to the radial shrinkage of the microfibril network. After the hot-water stretching stage (WP3), the void width further reduces to 24.27 nm, while the length increases to 42.38 nm, surpassing its width. This reversal indicates substantial microfibril rearrangement and merging during stretching. The subsequent drying densification process (WP4) significantly reduces the void width to 4.71 nm, while the length slightly increases to 47.89 nm, indicating the formation of narrow voids. Ultimately, steam stretching (WP5) extends the void length to 222.54 nm. In addition, the void length in WP5 is shorter than that in DP5, and its *B_eq_* value (13.9°) is obviously higher than that of DP5 (5.2°). This result suggests that WP5 has a greater number of inter-fibrillar binding sites and a lower degree of oriented arrangement of fibrils compared to DP5.

### 3.3. Crystallinity and Orientation

**Figure 5:**
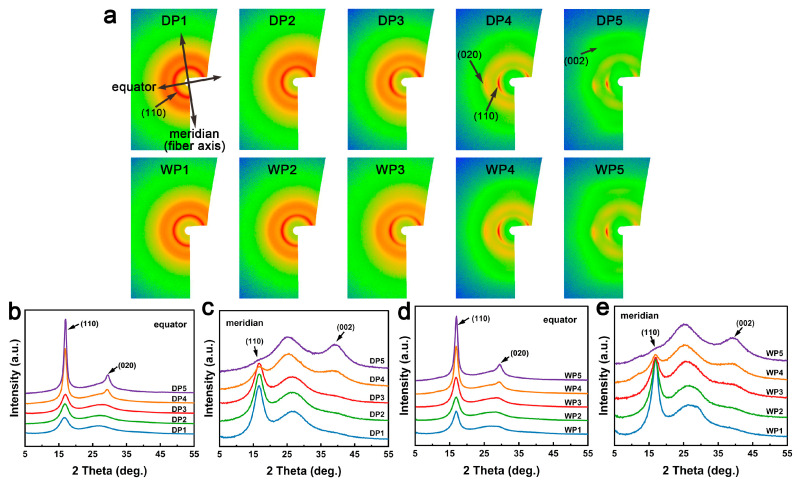
(**a**) Two-dimensional WAXS patterns of PAN fibers at key stages in the dry-jet wet-spinning process (DP1–DP5) and wet-spinning process (WP1–WP5). One-dimensional WAXS curves for dry-jet wet-spun fibers (equatorial: (**b**); meridional: (**c**)); and wet-spun fibers (equatorial: (**d**); meridional: (**e**)).

The WAXS patterns of PAN fibers during various stages of the wet-spinning and dry-jet wet-spinning processes are illustrated in Figure 5. The 2D WAXS patterns for DP1 and WP1 (Figure 5a) exhibit two diffuse diffraction rings at 2*θ* ≈ 16.9° (corresponding to the (110) reflection of the orthorhombic crystal model [34]) and a range of 2*θ* from 20° to 35°, respectively. The 1D WAXS curves of DP1 and WP1 display peaks around 16.9° with broad shapes and low intensities (Figure 5b–e), indicating the presence of numerous imperfect crystallites with low orientation in the two nascent fibers. During the post-spinning process, the diffused diffraction rings in 2D WAXS patterns (Figure 5a) evolve into two pairs of symmetrical arcs (2*θ* ≈ 16.9°, 29.5°) in the equator direction, corresponding to the (110) and (020) reflections, respectively. Additionally, a new weak scattering signal emerges at 2*θ* ≈ 40° in the meridional direction, corresponding to the (002) reflection, and some off-axis scattering present at 2*θ* ≈ 26°, corresponding to interference of nitrile groups within the oriented molecular chains [34,35]. For the equatorial 1D WAXS curves (Figure 5b,d), the peaks at 2*θ* ≈ 16.9° and 29.5° sharpen gradually. In contrast, for the meridional curves (Figure 5c,e), the peak at 2θ ≈ 16.9° gradually disappears, while the weak scattering at 2*θ* ≈ 40° intensifies. These results indicate that the alignment of the molecular chains improves and that the growth and orientation of the crystals occur during the post-spinning processes. These changes are particularly prominent during the drying densification stage, suggesting that this stage is critical for crystal evolution.

**Figure 6:**
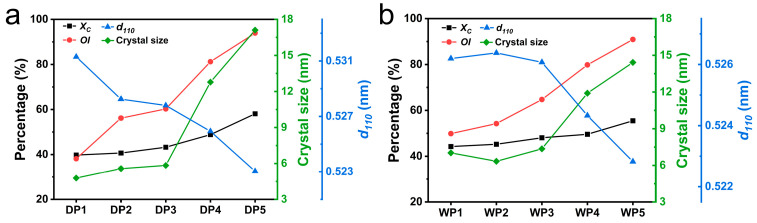
The crystallinity (*X_C_*), crystal orientation index (*OI*), crystal size, and crystal plane spacing (*d_110_*) of PAN fibers at key stages during the dry-jet wet-spinning process (**a**) and wet-spinning process (**b**).

The radial crystal structure parameters of PAN fibers during the two spinning processes are analyzed using equatorial curves, as shown in Figure 6. Detailed parameters of WAXS peak fitting are provided in Figures S1–S10 and Tables S1 and S2 in the Supplementary Materials. The results indicate that crystalline increases during both processes. Notably, small crystal sizes and large crystal plane spacings are maintained during the coagulation bath, washing, and hot-water stretching stages. After the drying densification stage, the crystal size increases significantly, while the crystal plane spacing decreases significantly. Subsequently, steam stretching further promotes the increase in crystal size and the decrease in crystal plane spacing. Additionally, from the coagulation bath to the steam stretching stage in both spinning techniques, the crystal orientation index exhibits a consistent increase. Liu et al. [36] had noted that the stretching process is accompanied by the unkinking and straightening of polymer chains. Moskowitz et al. [24] revealed that as the tensile strain increases, the gauche conformation of PAN chains transitions to a trans-conformation. This transition results in an extended planar zigzag arrangement, which allows adjacent chains to align and condense, thereby forming larger ordered regions. In addition, large crystal plane spacing also occurs in solvated crystals [37], when solvent/water molecules are present in the PAN fibers. Therefore, during the coagulation bath, washing, and hot-water stretching stages, the kinks in PAN chains and the swelling effect caused by the solvent/water molecules disrupt the local order of PAN chains, resulting in crystal characteristics with a small size and large crystal plane spacing. High hot rolling temperatures during the drying densification stage facilitate rapid evaporation of moisture and trigger conformational changes and recrystallization of the PAN chains. Subsequently, the unkinking and tight arrangement of PAN chains further promote the increase in crystal size and the decrease in crystal plane spacing, under the synergistic effects of high-temperature steam plasticization and stretching. Ultimately, from DP1 to DP5, the crystallinity increases from 39.8% to 58.1% (increased by 46%) and the crystal size increases from 4.78 nm to 17.1 nm (increased by 258%), while the orientation index increases from 38.1% to 93.9% (increased by 146%). Similarly, from WP1 to WP5, the crystallinity increases from 44.2% to 55.5% (increased by 26%), the crystal size increases from 7.03 nm to 14.42 nm (increased by 105%), and the orientation index increases from 49.9% to 90.9% (increased by 82%). These changes indicate that the increase in the crystallinity, crystal size, and orientation index of the fibers during the dry-jet wet-spinning process is more pronounced than that during the wet-spinning process.

### 3.4. Thermomechanical Transitions

**Figure 7:**
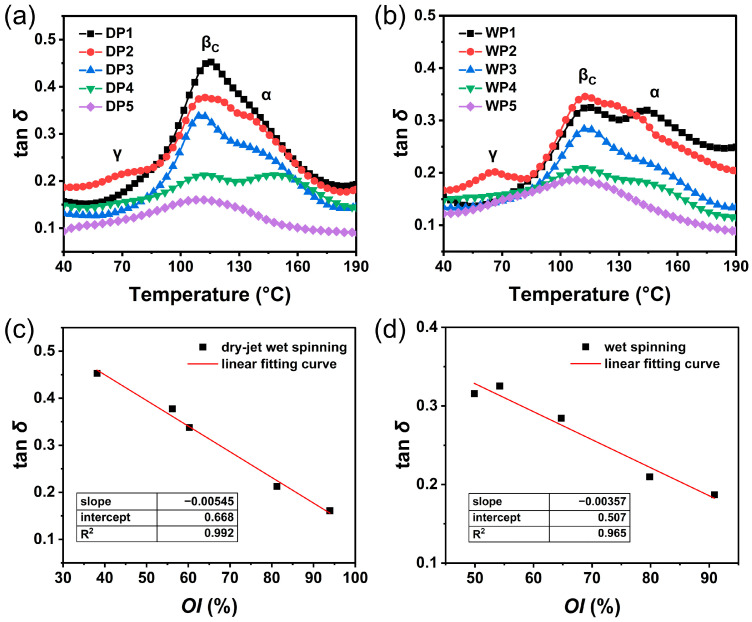
The tan *δ* curves of PAN fibers at key stages during dry-jet wet-spinning process (**a**) and wet-spinning process (**b**). The correlation between tan *δ* corresponding to βc transition and crystal orientation index (*OI*) for dry-jet wet-spinning process (**c**) and wet-spinning process (**d**).

The molecular chains in PAN fibers exist in both disordered and ordered states, which significantly influence the fibers’ viscoelastic behavior. These characteristics can be analyzed through the mechanical loss (tan *δ*) measured by DMA. Figure 7a,b show the evolution of tan *δ* in PAN fibers at key stages of both spinning processes. For nascent fibers, both DP1 and WP1 display two peaks in the tan δ curves. The α peak at higher temperature (approximately 145 °C) corresponds to the glass transition associated with molecular motion in the amorphous region, while the βc peak at lower temperature (approximately 113 °C) is attributed to the motion within the crystalline region, indicating the presence of a two-phase structure in the fibers [24,38,39]. Notably, the βc peak is significantly higher than the α peak for DP1, with the α peak appearing as a weak shoulder peak. As the temperature increases, the movable structural units within the PAN molecular chains gradually expand, leading to enhanced chain mobility. The synergistic effect of molecular relaxation in the imperfect, small-sized, and low-oriented crystals in the DP1 and the relaxation of short chain segments in the amorphous region contributes to the increased mechanical loss at the βc transition [40]. In comparison, the height of the βc peak is close to that of the α peak for WP1, attributed to the presence of larger crystal sizes and a greater orientation index in WP1 than those in DP1.

During the post-spinning processes, the heights of the transition peaks for the two series of PAN fibers show an overall decreasing trend. Notably, the heights of the two transition peaks for DP4 and WP4 exhibit a significant decline after the drying densification stage. This reduction can be attributed to the elevated roll temperatures (typically exceeding the βc transition temperature), which facilitate the removal of water molecules from the fibers and promote crystal growth. As the fibers underwent steam stretching, the α-transition peak disappears, and the height of the βc peak further diminishes, indicating a transition towards a single-phase structure. This phenomenon results from the increased mobility of PAN chains at high steam temperatures (generally approaching or exceeding its α transition temperature). The concurrent stretching effect further promotes the alignment of disordered chains and the growth of crystals. Ultimately, a single-phase structure is formed in both PAN precursor fibers, which corresponds to a state of thermodynamic equilibrium [38,40].

A comparison of the crystal orientation index of the fibers and the height of the βc peak revealed a clear correlation, as illustrated in Figure 7c,d. There is a gradual decrease in tan δ with an increasing orientation index during the two post-spinning processes. Additionally, tan *δ* (an indicator for material’s viscoelastic properties) is the ratio of the loss modulus to the storage modulus, and its decline signifies an enhancement in the elastic character of the PAN fibers. This change reveals the evolutionary relationship between the orientation index and the viscoelasticity of the fibers. The presence of small-sized imperfect crystals and disordered chains increases the viscous character of the fibers during the initial stages of the spinning process. Conversely, as a highly oriented and regularly arranged molecular structure develops within the fibers, the crystallinity, crystal size, and orientation index increase, thereby promoting a gradual improvement in elastic character during the post-spinning processes. Furthermore, comparison of Figure 7c,d shows that the decreasing slope is larger for the dry-jet wet-spinning process than for the wet-spinning process. This result indicates that the elastic nature of fiber responds more strongly to changes in the crystal orientation index during the dry-jet wet-spinning process, compared to the wet-spinning process. This difference corresponds to the more pronounced increase in the crystal size and orientation index of the fibers during the dry-jet wet-spinning process (Figure 6). These phenomena are mainly attributed to two factors: (1) the total drawing ratio applied during the dry-jet wet-spinning process is higher than that in the wet-spinning process; (2) the radial homogenous structure in dry-jet wet-spun fibers is beneficial for responding to the synergistic effects of the temperature and force fields and facilitates crystal development during post-spinning processes. In comparison, the skin-core structure in wet-spun fibers compromises structural integrity, which potentially limits synchronous radial improvements in fiber crystal structure under temperature and force fields during post-spinning processes.

### 3.5. Mechanical Properties

**Figure 8:**
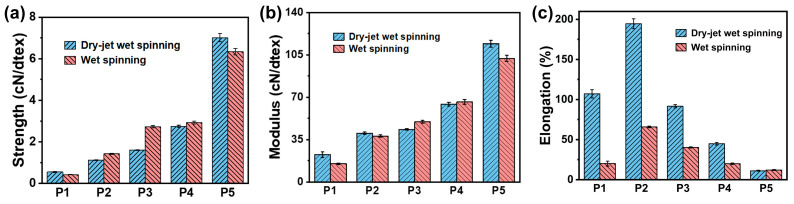
Mechanical properties of PAN fibers: (**a**) strength, (**b**) modulus, and (**c**) breaking elongation.

Figure 8 shows the mechanical properties of PAN fibers. As the spinning process progresses, the tensile strength of dry-jet wet-spun fibers increases significantly from 0.55 cN/dtex to 7.01 cN/dtex, while wet-spun fibers show a comparable enhancement from 0.43 cN/dtex to 6.34 cN/dtex (Figure 8a). Similarly, the modulus of the dry-jet wet-spun fibers rises from 22.6 cN/dtex to 114.3 cN/dtex, with wet-spun fibers exhibiting a parallel trend from 15.0 cN/dtex to 102.2 cN/dtex (Figure 8b). Based on comprehensive microstructural analysis, the enhancement in the mechanical properties of the fibers can be attributed to the gradual increase in crystal size, the progressive improvement in the crystallinity and orientation index, the optimized fibrous structure, and the reduced void defects [20,21,22]. The breaking elongation for both series of fibers initially increases from P1 to P2, followed by a gradual decrease from P2 to P5 (Figure 8c). In the nascent fibers, the loose microfibril network contracted after washing. However, their low molecular orientation persists, resulting in an increase in breaking elongation. Subsequent treatments, including hot-water stretching, drying densification, and steam stretching, improve molecular orientation in fibers, thereby reducing the breaking elongation progressively. Notably, from P1 to P4, the dry-jet wet-spun fibers exhibit significantly higher breaking elongation than wet-spun fibers (Figure 8c). This discrepancy probably arises from the large voids, skin-core structure, and the surface grooves of the wet-spun fibers, which induce stress concentration and accelerate crack initiation and breakage. As the post-spinning process proceeds, the difference in breaking elongation between the two types of fibers gradually decreases. In the final stage (DP5 and WP5), the breaking elongation of both fibers reaches a similar value. This phenomenon may occur because the void defects in the core region of the wet-spun fibers are gradually reduced, thereby making their mechanical properties more comparable to those of the dry-jet wet-spun fibers. Ultimately, the dry-jet wet-spun precursor fibers demonstrate similar breaking elongation but superior strength and modulus compared to the wet-spun precursor fibers. This enhancement can be attributed to their larger crystal size, higher crystallinity and orientation index, better fibrillar alignment, and fewer structural defects [15,17,18].

### 3.6. Structural Evolution Sketch

**Figure 9:**
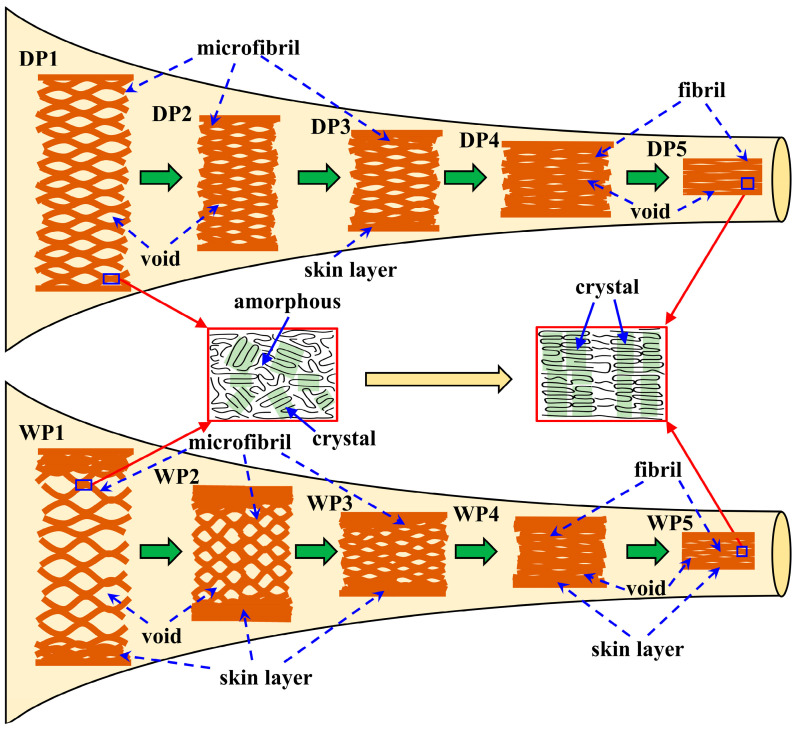
Structural evolution sketch of PAN fibers during dry-jet wet-spinning processes (DP1–DP5) and wet-spinning processes (WP1–WP5).

Figure 9 shows the structural evolution sketch of the PAN fibers during the two spinning processes. The structural evolution in both spinning processes exhibits several significant commonalities. In the coagulation bath stage, the nascent fibers exhibit a loose microfibril network with disordered crystalline arrangements. Subsequent stages including washing and hot-water stretching facilitate the gradual contraction and amalgamation of the microfibril network, accompanied by a reduction in void size. These structural changes result from the synergistic effects of temperature, mechanical tension, and water plasticization. Notably, the microfibril network contracts radially from the outer layer of the fibers, forming a dense skin layer. Meanwhile, crystal orientation progressively improves, whereas crystal size shows slightly fluctuation. After the drying densification process, the void size within the fibers decreases significantly, accompanied by both a reduction in moisture content and the merging of numerous microfibrils. Moreover, the elevated temperature and applied force field create favorable conditions for the β_c_ transition of the fiber, which facilitates the development of the crystals. Steam stretching further improves fibril alignment, crystal orientation, and the regularity of molecular arrangement. This process culminates in a thermodynamically stable single-phase structure.

However, the initial structure of PAN fibers, which is determined by their spinning processes, greatly influences the evolution of their microstructure. PAN nascent fibers (DP1) produced through the dry-jet wet-spinning process exhibit a uniformly distributed microfibril network and a smooth surface. Although the hot-water stretching causes the core-skin structure in the fibers, this heterogeneous structure gradually diminishes during subsequent drying densification and steam stretching processes. Ultimately, the resultant precursor fiber presents a relatively homogeneous fibrillar network and maintains a smooth surface. In contrast, the PAN nascent fibers (WP1) prepared using the wet-spinning method display a smooth surface but exhibit radial heterogeneity (skin-core structure) in the microfibril network. Notably, large voids exist in their core region. As the sparse microfibril network undergoes radial merging in the post-spinning process, deep grooves concurrently form on the fiber surface. The skin-core structure persists throughout the post-spinning process and remains in the final precursor fibers. Furthermore, compared to wet-spun nascent fibers, dry-jet wet-spun nascent fibers exhibit smaller crystal sizes and a lower crystal orientation index. Nevertheless, during the post-spinning process, these fibers experience a more significant increase in both the crystal size and orientation index. This improvement benefits from fewer structural defects and matching process conditions.

## 4. Conclusions

In this study, we compared the evolution of the microstructure and mechanical properties of PAN fibers between the wet-spinning and dry-jet wet-spinning processes. Both spinning processes exhibit a transformation from a loose microfibril network to an oriented arrangement of microfibrils and fibrils. Meanwhile, crystal alignment and molecular chain regularity are enhanced under tensile and temperature fields. Notably, the drying densification stage is a critical stage in the evolution of crystals and the enhancement of microfibrillar compactness. Moreover, along with the optimization of these microstructures, the fibers transition from a two-phase structure to a thermodynamically stable single-phase structure, and their elastic character, strength, and modulus are progressively improved. The breaking elongation follows an evolution trend that initially increases and then gradually decreases, with the washing stage as the critical point.

However, the radial structures of the nascent fibers prepared by these two processes show significant differences, which lead to some different characteristics of structural and mechanical evolution during post-spinning processes. PAN nascent fibers manufactured by wet spinning exhibit inherent structural defects, including a significant skin-core structure and large voids in the core region. These defects lead to the formation of surface grooves and significant radial mechanical differences during subsequent post-spinning processes, which further compromise the mechanical properties of the fibers. In contrast, PAN nascent fibers prepared by dry-jet wet spinning possess a smooth surface and a uniformly distributed microfibrillar network. These favorable structural characteristics evolve during post-spinning processes into well-oriented and radially homogeneous fibrillar structures. Moreover, the dry-jet wet-spinning process shows more pronounced increases in crystallinity (46%), crystal size (253%), and orientation index (146%), compared to the wet-spinning process. Ultimately, the dry-jet wet-spun PAN precursor fibers exhibit superior fibrillar alignment, a larger crystal size (16.7 nm), a higher crystallinity (58.1%) and orientation index (93.9%), fewer structural defects, and better mechanical properties (tensile strength: 7.01 cN/dtex; modulus: 114.3 cN/dtex), compared to wet-spun PAN precursor fibers. The dry-jet wet-spinning process offers significant advantages in forming and optimizing microstructure of the fibers, thus demonstrating greater potential for producing high-quality PAN precursor fibers.

## Data Availability

Data are contained within the article or Supplementary Materials. Further inquiries can be directed to the corresponding author.

## Supplementary Materials

The following supporting information can be downloaded at https://www.mdpi.com/article/10.3390/polym17182504/s1. Figure S1: WAXS peak fits for DP1; Figure S2: WAXS peak fits for DP2; Figure S3: WAXS peak fits for DP3; Figure S4: WAXS peak fits for DP4; Figure S5: WAXS peak fits for DP5; Figure S6: WAXS peak fits for WP1; Figure S7: WAXS peak fits for WP2; Figure S8: WAXS peak fits for WP3; Figure S9: WAXS peak fits for WP4; Figure S10: WAXS peak fits for WP5; Figure S11: The azimuthal intensity data of the (110) crystal plane: dry-jet wet-spun fibers (a) and wet-spun fibers (b); Table S1: Parameters of WAXS peak fitting for DP1–DP5; Table S2: Parameters of WAXS peak fitting for WP1–WP5.
