# Supplementary material for: Evolution of Microstructure and Performance in Polyacrylonitrile Precursor Fibers: A Comparison of Spinning Processes
Source: Polymers (Basel). 2025 Sep 17;17(18):2504. doi: 10.3390/polym17182504 (PMC12473616; doi:10.3390/polym17182504)
Supplement: Supplementary file 1 [file polymers-17-02504-s001.zip › polymers-3842776-supplementary.pdf]

Supporting Materials

Evolution of Microstructure and Performances in Polyacrylonitrile Precursor Fibers: A Comparison of Spinning Processes

Liang Cao [1,2,3], Lili Zhang [1,3], Zhenbo Zhao [1,3], Shaowei Wang [1,2,3], Zhaowei Li [1,2,3], Deqi Jing [1,2,3,*] and Shouchun Zhang [1,2,3,*]

1 Research Center of Advanced Thermoplastic Composites Engineering, Institute of Coal Chemistry, Chinese Academy of Sciences, Taiyuan 030001, China

2 Center of Materials Science and Optoelectronics Engineering, University of Chinese Academy of Sciences, Beijing 100049, China

3 Shanxi Key Laboratory of Carbon Materials, Institute of Coal Chemistry, Chinese Academy of Sciences, Taiyuan 030001, China

* Correspondence: jingdq@sxicc.ac.cn (D.J.); zschun@sxicc.ac.cn (S.Z.)

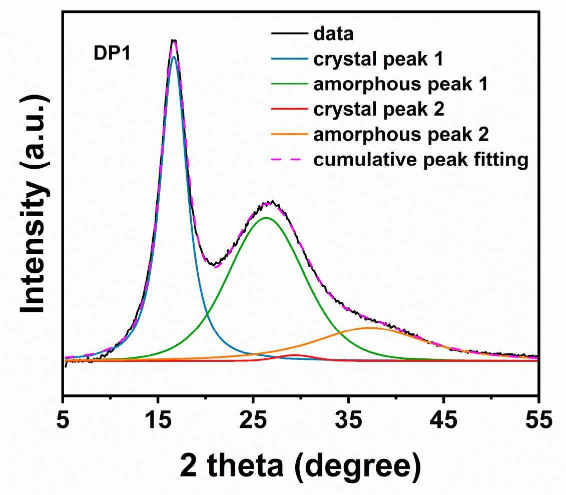

Figure S1. WAXS peak fits for DP1.

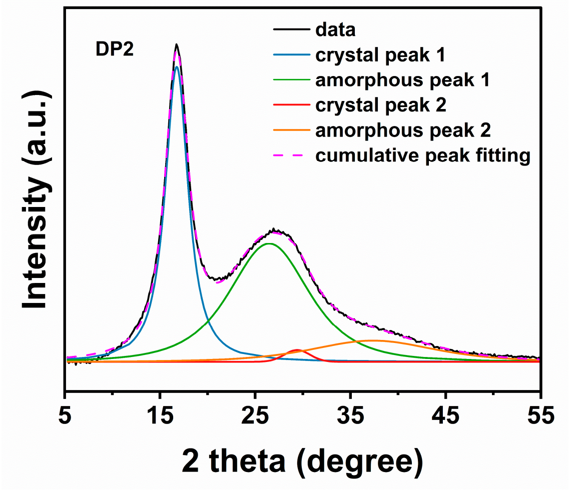

Figure S2. WAXS peak fits for DP2.

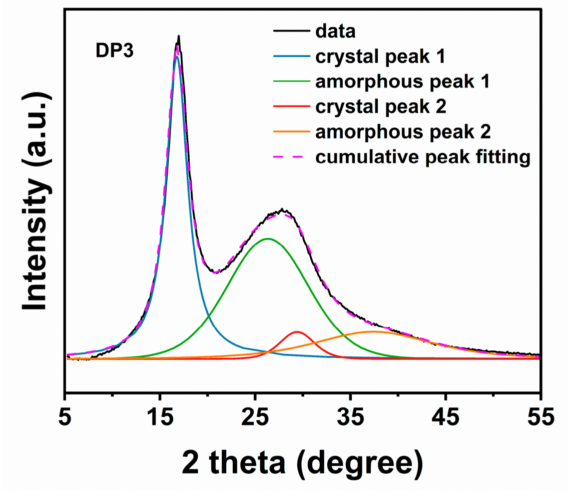

Figure S3. WAXS peak fits for DP3.

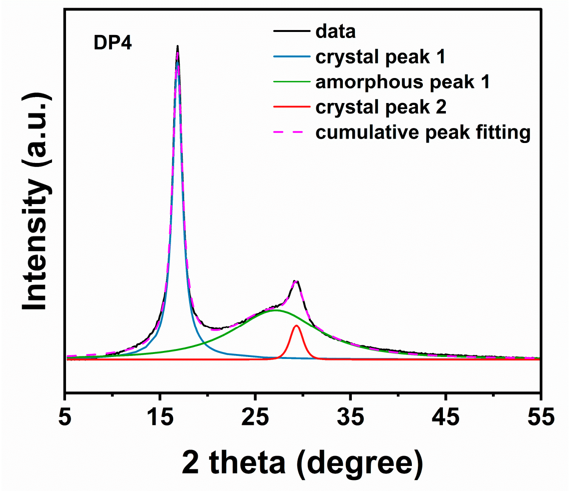

Figure S4. WAXS peak fits for DP4.

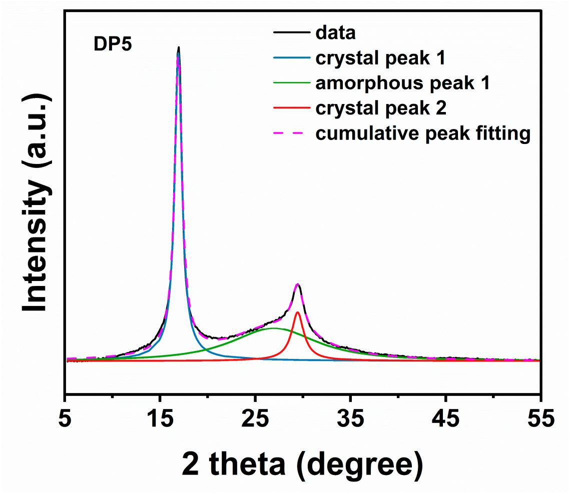

Figure S5. WAXS peak fits for DP5.

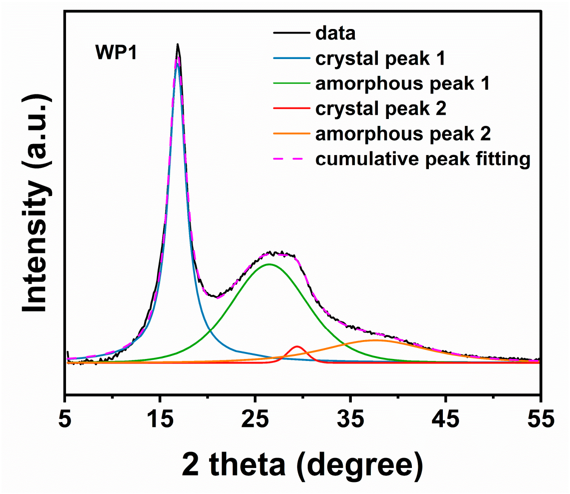

Figure S6. WAXS peak fits for WP1.

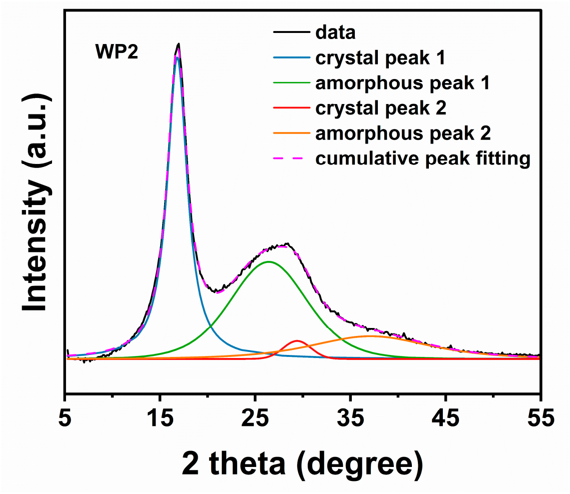

Figure S7. WAXS peak fits for WP2.

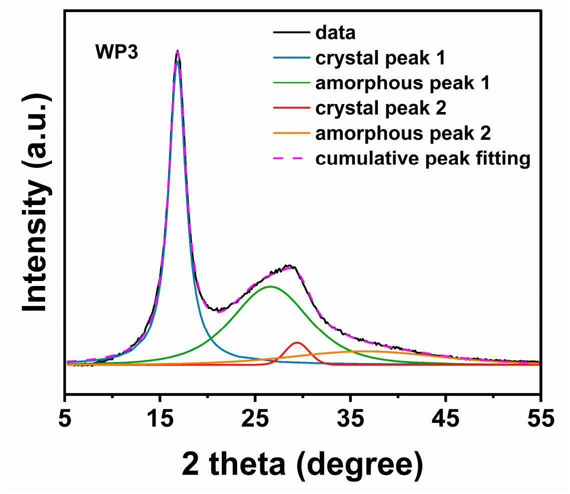

Figure S8. WAXS peak fits for WP3.

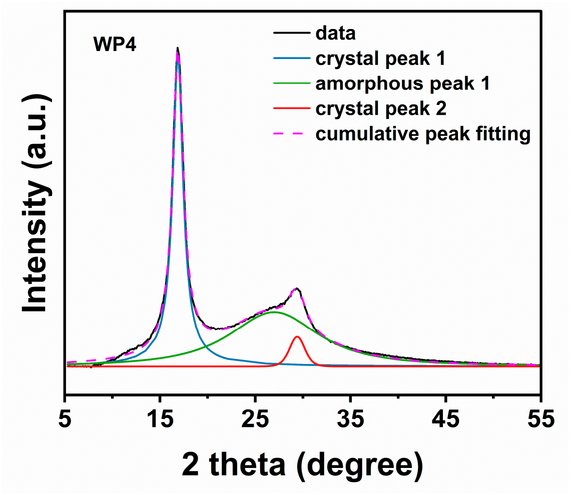

Figure S9. WAXS peak fits for WP4.

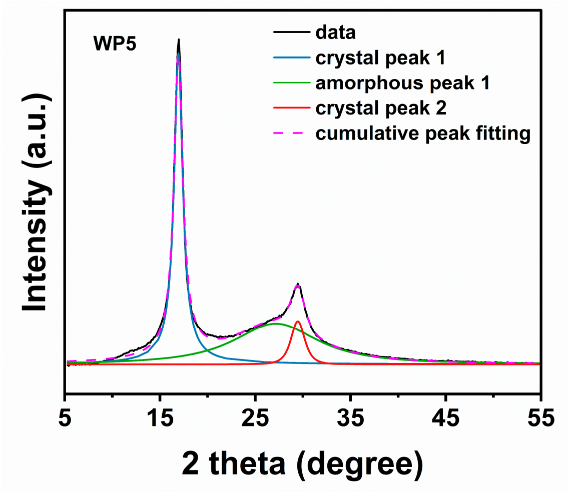

Figure S10. WAXS peak fits for WP5.

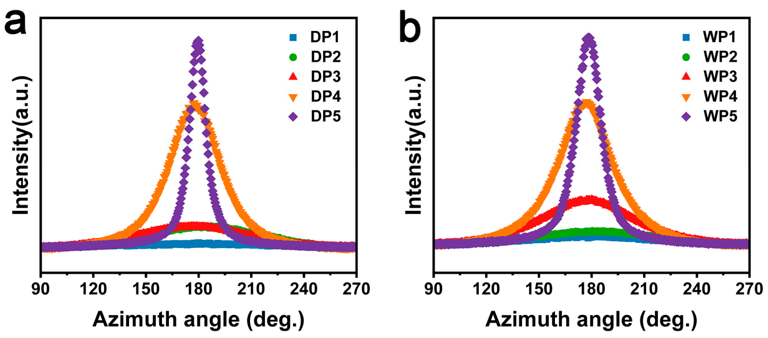

Figure S11. The azimuthal intensity data of the (110) crystal plane: dry-jet wet-spun fibers (a) and wet-spun fibers (b).

Table S1. Parameters of WAXS peak fitting for DP1–DP5.

Parameters of peak fitting		DP1	DP2	DP3	DP4	DP5
Crystal peak 1	2 Theta (Deg.)	16.666	16.763	16.779	16.835	16.931
	FWHM (Deg.)	3.360	2.881	2.749	1.257	0.939
	Area	0.01929	0.01995	0.01340	0.01814	0.01491
Amorphous peak 1	2 Theta (Deg.)	26.371	26.469	26.332	27.157	26.964
	FWHM (Deg.)	9.505	9.878	10.074	10.799	10.601
	Area	0.02190	0.02380	0.00534	0.02095	0.01351
Crystal peak 2	2 Theta (Deg.)	29.401	29.405	29.402	29.331	29.456
	FWHM (Deg.)	4.549	3.174	4.251	1.575	1.569
	Area	0.00039	0.00067	0.00119	0.00184	0.00383
Amorphous peak 2	2 Theta (Deg.)	37.252	37.369	37.419	--	--
	FWHM (Deg.)	13.552	15.111	14.436	--	--
	Area	0.00788	0.00629	0.00120	--	--
R^2		0.9985	0.9986	0.9961	0.9977	0.9971

Table S2. Parameters of WAXS peak fitting for WP1–WP5.

Parameters of peak fitting		WP1	WP2	WP3	WP4	WP5
Crystal peak 1	2 Theta (Deg.)	16.829	16.823	16.833	16.889	16.938
	FWHM (Deg.)	2.285	2.532	2.181	1.349	1.114
	Area	0.01680	0.01599	0.01684	0.02481	0.04740
Amorphous peak 1	2 Theta (Deg.)	26.491	26.425	26.599	26.987	27.165
	FWHM (Deg.)	9.499	9.546	9.525	11.099	10.503
	Area	0.01652	0.01492	0.01484	0.02744	0.04542
Crystal peak 2	2 Theta (Deg.)	29.405	29.403	29.405	29.406	29.471
	FWHM (Deg.)	2.397	3.435	2.959	1.884	1.686
	Area	0.00066	0.00094	0.00118	0.00219	0.00718
Amorphous peak 2	2 Theta (Deg.)	37.627	37.103	36.525	--	--
	FWHM (Deg.)	13.041	14.590	18.249	--	--
	Area	0.00549	0.00558	0.00464	--	--
R^2		0.9971	0.9985	0.9988	0.9979	0.9971
